# Use of the Internet by Women with Breast Cancer

**DOI:** 10.2196/jmir.4.2.e9

**Published:** 2002-11-22

**Authors:** Joshua Fogel, Steven M Albert, Freya Schnabel, Beth Ann Ditkoff, Alfred I Neugut

**Affiliations:** ^1^Queen Elizabeth II Health Sciences CentreDepartment of PsychologyHalifaxNova ScotiaCanada; ^2^College of Physicians and SurgeonsColumbia UniversityDepartment of NeurologyNew York NYUSA; ^3^College of Physicians and SurgeonsColumbia UniversityDepartment of SurgeryNew York NYUSA; ^4^College of Physicians and SurgeonsColumbia UniversityDepartment of MedicineNew York NYUSA; ^5^College of Physicians and SurgeonsColumbia UniversitySchool of Public HealthNew York NYUSA; ^6^College of Physicians and SurgeonsColumbia UniversityGertrude H. Sergievsky CenterNew York NYUSA; ^7^College of Physicians and SurgeonsColumbia UniversityHerbert Irving Comprehensive Cancer CenterNew York NYUSA

**Keywords:** breast cancer, communication, decision-making, information, Internet

## Abstract

**Background:**

Recently, many cancer patients have been using the Internet for information with which to make informed choices. We are not aware of any studies that investigate this Internet use among breast cancer patients or women.

**Objective:**

We investigate the prevalence and predictors of Internet use for medical information among women with breast cancer.

**Methods:**

We used a cross-sectional design and approached 251 women with breast cancer being treated at a university-based hospital. We successfully interviewed 188 (74.9%), through mailed self-report questionnaires. Medical information was obtained from the hospital tumor registry. We used t tests and chi-square tests to assess differences in Internet use for breast health issues and binary logistic regression to estimate the odds ratio (OR) for predictors of Internet use for breast health issues.

**Results:**

In our sample, 41.5% of patients used the Internet for medical information. Internet users differed from nonusers on income level, educational level, and by race/ethnicity. After controlling for the other predictors, Internet users had a higher income (OR = 3.10; 95% CI = 1.09-8.85) and tended to be more educated (OR = 2.59; 95% CI = 0.87-7.74) than nonusers. There was also a suggestion that those of nonwhite ethnicity were less likely to use the Internet (OR = 0.39; 95% CI = 0.14-1.11). Increasing age, length of time since diagnosis, and breast cancer stage had no effect.

**Conclusions:**

A substantial proportion of breast cancer patients used the Internet as a source of information. Patients with higher income or education, and patients of white race/ethnicity are more likely to use the Internet for breast health issues.

## Introduction

Physicians have traditionally been the sole providers of information to patients about their diagnosis, prognosis, and treatment options. Until recently, many physicians believed that patients could not cope with bad news and should be kept ignorant of many details about their illness [1].

Many patients no longer feel comfortable with this paternalistic approach and are becoming more insistent about being fully informed and participating in their treatment decision-making. Some studies of cancer patients indicate patient preferences for knowing as much as possible, ranging from 79% to 96% [2-6]. These information seekers tend to be of higher socioeconomic status, younger age, and white race/ethnicity [2,4] and are more hopeful about their prognosis [2,7]. Those in the US are more likely to seek information in a variety of areas than those from the UK [8].

Cancer patients are often dissatisfied with the information provided to them. One study showed that only 19% of 232 patients were satisfied with the information they received from their physicians [9]. Studies done specifically with breast cancer patients show that many want to have a collaborative role with their physician in major treatment decisions [10,11] and many desire more detailed information [12].

Patients use the Cancer Information Service (National Cancer Institute; http://cis.nci.nih.gov/), printliterature, television, and radio for information [13,14]. Whites are more likely to rely on books, while African-Americans are more likely to use television and radio programs as sources of information [14]. One new source of cancer information is the Internet. It is widely available; individuals can access it at work, home, and their local libraries. Physicians themselves are increasingly using it for information [15,16]; 20% consider its use essential to their duties as a physician [17].

Although there are potential risks for use of the Internet because the information is unmonitored [18-20], patients are increasingly turning to it for information. A 1997 survey in the US found that nearly half of Internet users spent some time looking for health information on the Internet [21]. In the US in 2000, 41 million individuals [17] and — a survey conducted in the US in March 2001 suggested — 100 million individuals [22] were estimated to have sought health information on-line. Patients report that Internet use often keeps them more informed than the doctors to whom they go for treatment [23]. Cancer is 1 of the top 3 diseases about which the public seeks information on the Internet [24].

Few studies have explored the use of the Internet by cancer patients and — to our knowledge — below are reviewed all the studies. One qualitative study evaluated a computer-based cancer-support network for individuals coping with cancer [25]. The only demographic characteristics mentioned were gender and marital status. Another qualitative study evaluated participants of an on-line breast cancer listserv [26] and did not provide the demographic characteristics of the participants. A Swedish study of 142 cancer patients found that only 8 (6%) used the Internet for information [27]. A recent Canadian descriptive study of mixed-diagnosis cancer patients found that 51% searched the Internet for medical information [28]. Another recent study discussed Internet use by prostate cancer patients and found users more likely to be younger, more educated, to own a personal computer, and to have prior experience with computers [29]. A recent preliminary Norwegian study of 31 cancer patients found that 4 (13%) used the Internet for medical information. These Internet users were slightly younger than nonusers were [30].

This is the first study that we are aware of both breast cancer patients and women who use the Internet for medical information. We investigate the prevalence and predictors of Internet use by women with breast cancer for information related to breast health issues.

## Methods

The participants for this study were patients seen by 2 breast surgeons at Columbia Presbyterian Medical Center, a university-based hospital in New York City. Inclusion criteria included age < 65 years and a diagnosis of ductal carcinoma in situ (DCIS) or invasive breast cancer within 3 years. All patients who met these criteria were invited to participate. Participants with a prior psychiatric/substance abuse history or who did not speak English were excluded. Institutional review board approval was obtained.

Participants were identified from hospital tumor-registry records. Potential participants were mailed a letter describing the study along with a postal card to return if they were not interested in participating. Those who did not return the postal card were called and the nature of the study described. Those who agreed to participate were mailed a packet with a questionnaire containing demographic and Internet-use questions. The Internet-use questions asked participants to circle *yes* or *no* to the question, "Do you use the Internet?" If *yes*, they were asked to circle locations of use (home, work, library, friend). For our study, we defined Internet use as World Wide Web use for information regarding breast health/women's health issues. We determined such use by asking participants to circle *yes* or *no* to the question, "Do you use the world wide web?" If *yes*, they were asked, "Do you use it for information regarding breast health/women's health issues?" See Appendix for the Internet use questionnaire used in this study.

A postage-paid envelope was provided. If necessary, two follow-up phone calls were made to remind participants. We approached 251 individuals (including 18 who initially declined, 25 who declined after any of the phone calls, and 20 who did not return their questionnaires). Of the 251, 188 (74.9%) chose to participate. Medical information was obtained from hospital tumor-registry records. All data collection took place from October to December 2000. Informed consent was obtained.

## Data Analysis

Our analyses compared users with nonusers of the Internet for breast health issues. T tests for independent samples were used to evaluate differences for continuous demographic variables and chi-square analyses assessed group differences for categorical variables. All categorical variables were dummy coded for inclusion in a regression analysis. African-Americans and Hispanic-Americans were combined into nonwhites in the race/ethnicity category for all analyses. The 7 Asians and 1 unidentified in the race/ethnicity category were excluded from the regression analysis due to their small number. The primary analysis used binary logistic regression to determine odds ratios for Internet use, controlling for the other predictors. All *P* values were 2-sided. All analyses were done with SPSS (Version 9) [31].

## Results

Table 1 describes the characteristics of users and nonusers of the Internet (ie, World Wide Web) for breast health. In our sample, 41.5% used the Internet. Sources of Internet use were at home (53.7%), at work (35.1%), at a friend's house (5.9%), and at a library (5.3%). With univariate analyses, Internet users were more educated, of higher income, more likely to be white, had a trend to be younger, and differed neither in breast cancer stage nor in length of time since their cancer diagnosis.

**Table 1:** Characteristics of 188 women with breast cancer*

Demographic Variable	Category	Web Use Mean (SD) / # (%) (N = 78)	No Web Use Mean (SD) / # (%) (N = 110)	Significance (*P*)‡
Age (years)		50.21 (7.69)	52.35 (8.71)	.08
Time since diagnosis (years)		1.75 (0.80)	1.93 (0.81)	.13
Annual household income	< $60,000	9 (12.7%)	36 (37.5%)	.001
	$60,000-$100,000	26 (36.6%)	30 (31.3%)	
	> $100,000	36 (50.7%)	30 (31.3%)	
Education	Grades < 12	9 (11.5%)	35 (32.1%)	.004
	Grades 13-16	35 (44.9%)	40 (36.7%)	
	Grades > 16	34 (43.6%)	34 (31.2%)	
Race/ethnicity	White	66 (86.8%)	77 (74.0%)	.04
	Nonwhite	10 (13.2%)	27 (26.0%)	
Stage	DCIS	19 (24.7%)	25 (22.9%)	.96
	Stage 1	32 (41.6%)	47 (43.1%)	
	Stage 2-3	26 (33.8%)	37 (33.9%)	

^*^ From interviews at Columbia-Presbyterian Medical Center, October 2000 to December 2000, regarding Internet (World Wide Web) use for breast health Issues. Not all variables have the total N = 188 since not everyone responded to all the items on the self-report measures.

*P* values were calculated with t tests for the means and chi-square tests for the percentages.

**Table 2:** Predictors of Internet use of 188 women with breast cancer*

Demographic Variable	Category	OR‡	95% CI†	Significance (*P*)
Age (years)		0.97	0.92-1.02	.19
Time since diagnosis (years)		0.73	0.46-1.15	.18
Annual household income	< $60,000	1.00		
	$60,000-$100,000	2.81	1.00-7.91	.05
	> $100,000	3.10	1.09-8.85	.04
Education	<Grades < 12	1.00		
	Grades 13-16	2.92	1.00-8.54	.05
	Grades > 16	2.59	0.87-7.74	.09
Race/ethnicity	White	1.00		
	Nonwhite	0.39	0.14-1.11	.08
Stage	DCIS	1.00		
	Stage 1	0.94	0.38-2.34	.89
	Stage 2-3	1.95	0.73-5.21	.18

^*^ From interviews at Columbia-Presbyterian Medical Center, October 2000 to December 2000, regarding Internet (World Wide Web) use for breast health Issues. Not all variables have the total N = 188 since not everyone responded to all the items on the self-report measures. Logistic regression analysis performed, controlling simultaneously for the other predictors above.

^‡^ OR indicates odds ratio

^†^ CI indicates confidence interval

Table 2 shows the results of logistic regression analysis, controlling for the other predictors. The model was significant (χ ^2^= 27.67, *P*= .001). As can be seen, income level remained significantly related to Internet use, as did increased educational level. Those with an income level > $60,000 were 3 times more likely to use the Internet than people with incomes < $60,000. Patients with a college education (ie, those in the groups of grades 13-16 and > grade 16) were almost 3 times more likely to use the Internet than those with a high school education or less. Nonwhite patients were less likely to use the Internet than whites, but this did not reach statistical significance. Age, length of time since diagnosis, and breast cancer stage were unrelated to Internet use.

## Discussion

Internet use is popular among breast cancer patients. Over 40% of our sample used it for breast health issues. In addition, our results are consistent with the prior literature suggesting that higher income and race/ethnicity are associated with patient information seeking [2,4,14].

We found that increased income and educational level were significant predictors of Internet use. Individuals with these characteristics may have been exposed to newer technology and have the comfort level to experiment with Internet use. They also may be more likely to use the Internet as part of their daily work. Race/ethnicity is related to Internet use where whites use the Internet more than nonwhites do.

In a study of Internet use by patients with prostate cancer [29], income level was not assessed. Our study shows that income level is strongly associated with Internet use and is a significant predictor of use of the Internet by patients with serious illnesses.

In our study, age, length of time since diagnosis, and breast cancer stage were not significant predictors of Internet use. The absence of an age effect in our study may differ from other studies of information use because we excluded those > 65 years from the study. Our results have adequate sample size and answer many of the preliminary questions of Norum [30].

The strengths of our study include the high participation rate and the inclusion of those with different stages of disease. However, we relied on self-report and did not have a way of independently validating the reported use. Our sample included those of multiethnic populations. However, these results would be strengthened by having a greater percentage (eg, 50%) of participation by those from multiethnic populations.

Internet use may have clinical relevance. Eakin and Stryker [32] showed that 70% of physicians refer their cancer patients to various support services. Patient use of these services is quite low, ranging from 2% to 8%. Of those patients aware of Internet-based cancer information services, which they found to be 14%, one half (7%) used it. Many patients may find it more comfortable to seek information over the Internet than to use traditional cancer support services.

The generalizability of these findings may be limited to those with early-stage breast cancer, women < 65 years, higher income, higher education, and those with a diagnosis of almost 2 years. Although not deliberately screened out, there were no patients with stage 4 breast cancer. It is possible that many of these late-stage patients died during the time interval from diagnosis to study completion or refused to participate. For those recently diagnosed, improved mammography screening rates allow many to be diagnosed with an early-stage rather than a late-stage cancer. Furthermore, the participants were only selected from 2 surgeon's practices and the income and education may be higher than those with breast cancer in the general population in the US. This may limit the generalizability of this study and future studies should include other hospitals/health centers to determine if these results could generalize to all breast cancer populations in other regions or countries.

Longitudinal research should investigate Internet use among various stages and times since diagnosis among breast cancer patients. Time sampling of Internet use at various intervals in an objective manner can improve these self-report results. As elderly women become more comfortable with Internet use, their use should be studied. More knowledge is needed about the quality of the Web sites used, the types of information sought, and the involvement of Internet use for patient decision-making. Research should evaluate if patients and/or physicians feel there are potential clinical benefits for this Internet use. Part of the work reported in references [33-35] is based on information from the questionnaire in the Appendix.

## Abbreviations

CI: Confidence Interval
OR: Odds Ratio

## Appendix 1

Internet Use Questionnaire
