# Supplementary material for: Use of the Internet by Women with Breast Cancer
Source: J Med Internet Res. 2002 Nov 22;4(2):e9. doi: 10.2196/jmir.4.2.e9 (PMC1761930; doi:10.2196/jmir.4.2.e9)
Supplement: Supplementary file 1 [file jmir_v4i2e9_app1.doc]

Do you use the Internet (circle one) Yes No

If **no**, you are now finished

If **yes,** continue below:

Internet access (circle as many as relevant)

1. home
2. work
3. library
4. friend

Do you use the world wide web (circle one) Yes No

If yes, do you use it for information regarding breast health / women’s health issues? Yes No

If yes, estimated **current** number of hours weekly .

If yes, estimated number of hours weekly **before surgery** .

Do you use e-mail (circle one) Yes No

If yes, do you use it for information regarding breast health / women’s health issues? Yes No

If yes, estimated **current** number of messages weekly .

If yes, estimated number of messages weekly **before surgery** .

Are you part of a list serv (circle one) Yes No

If yes, do you use it for information regarding breast health / women’s health issues? Yes No

If yes, estimated **current** number of hours weekly .

If yes, estimated number of hours weekly **before surgery** .

Are you part of a news group / chat group (circle one) Yes No

If yes, do you use it for information regarding breast health / women’s health issues? Yes No

If yes, estimated **current** number of hours weekly .

If yes, estimated number of hours weekly **before surgery** .

Are you part of an Internet self-help / support group (circle one)

Yes No

If yes, do you use it for information regarding breast health / women’s health issues? Yes No

If yes, estimated **current** number of hours weekly .

If yes, estimated number of hours weekly **before surgery** .
